# Architectural degeneration and dysfunction of Cerebral vessels in a mouse model of Alzheimer's disease

**DOI:** 10.1002/alz70855_106697

**Published:** 2025-12-24

**Authors:** Moltira Promkan, Kewarin Jinawong, Rungruedee Kimseng, Kamonchat Phikulthong, Susan D Kraner, Colin B Rogers, Tiffany L. Sudduth, Erica M. Weekman, Jitbanjong Tangpong, Donna M. Wilcock, Peter T Nelson, Pradoldej Sompol

**Affiliations:** ^1^ University of Kentucky, Lexington, KY, USA; ^2^ University of Kentucky College of Medicine, Sanders‐Brown Center on Aging, Lexington, KY, USA; ^3^ University of Kentucky / Sanders‐Brown Center on Aging, Lexington, KY, USA; ^4^ Indiana University School of Medicine, Stark Neurosciences Research Institute, Department of Neurology, Indianapolis, IN, USA; ^5^ Walailak University, Thasala, Nakhon Si Thammarat, Thailand

## Abstract

**Background:**

Several forms of cerebrovascular pathology are highly comorbid with clinical hallmarks of Alzheimer's disease. These understudied pathological lesions could accelerate disease progression and affect treatment efficacy. Here, we aim to investigate whether aging and a genetic predisposition to Alzheimer's disease can worsen cerebrovascular architecture and function.

**Method:**

Cranial window surgery was performed for intravital two‐photon imaging to investigate vascular architecture, pathology, and function in the brains of young (3‐5 months) and old (24 months) wild‐type and Alzheimer's mouse model (Tg2576). Methoxy‐X04 was used to identify Aβ plaques and cerebral amyloid angiopathy (CAA), while rhodamine dextran was used to visualize the bloodstream. Air‐puff stimulation of contralateral whiskers was applied to induce penetrating arteriole dilation as a measure of neurovascular coupling in awake mice. Human brain sections were used as reference pathology. Immunochemistry and immunofluorescence were employed to confirm vascular pathology and abnormalities.

**Result:**

A reduction in neurovascular function was observed in aged wild‐type mice, and more severe impairment was found in vessels with CAA pathology in Tg2576 brains. Beta amyloid predisposition increased cortical leptomeningeal and penetrating vessel aneurysms, both blebbing and saccular. Microvessel tortuosity characteristics, including curved, looped, and folded vessels, were increased in aged wild‐type mice and extensively increased in Tg2576 mice. Cerebrovascular integrity, indicated by lectin staining, was reduced, and the astrocyte marker GFAP was increased in Tg2576 compared to wild‐type mice. These results highlight that the vascular pathology found in Tg2576 brains recapitulates the vascular pathology found in human brains.

**Conclusion:**

These results emphasized the impact of aging and Aβ predisposition on vascular architectural degeneration and dysfunction. These findings provide translational imaging approaches that could be used for further investigation of pathogenesis, pathophysiology and treatment development for AD and vascular‐related neurodegenerative diseases.

